# Combined CMR and catheterization data in determining right ventricular-arterial coupling in children and adolescents with pulmonary arterial hypertension

**DOI:** 10.1186/1532-429X-16-S1-O43

**Published:** 2014-01-16

**Authors:** Uyen Truong, Sonali Patel, Brian Fonseca, Jamie Dunning, Dunbar Ivy, Robin Shandas, Kendall Hunter

**Affiliations:** 1 Pediatric Cardiology, Children's Hospital Colorado, Aurora, Colorado, USA; 2 Bioengineering, University of Colorado Denver Medical Campus, Aurora, Colorado, USA

## Background

Pulmonary arterial hypertension (PAH) remains a disease with high morbidity/mortality in pediatrics. Understanding ventricular-arterial coupling, a measure of how well matched the ventricular and vascular function is, may elucidate the pathway leading to right heart failure.

## Methods

This retrospective study included subjects with PAH who a cardiac magnetic resonance (CMR) study within 14 days of cardiac catheterization between January 2009-August 2013. The effective elastance (Ea, index of arterial load) and right ventricular maximal end-systolic elastance (Emax, index of contractility) were determined by a combination of CMR and hemodynamic data. Ea is defined as (mean pulmonary arterial pressure minus pulmonary capillary wedge pressure)/stroke volume. Emax is defined as mean pulmonary arterial pressure/end systolic volume. Ea/Emax ratio was derived. Additionally, a measure of non-invasive ventricular arterial coupling (assuming PWCP is insignificant, making Ea/Emax = end systolic volume/stroke volume) was derived from only CMR. Pulmonary vascular resistance indexed (PVRi) and pulmonary vascular reactivity, as defined by Barst criteria (decrease in mean pulmonary artery pressure of > 20%, unchanged/increased cardiac index, and decreased/unchanged pulmonary to systemic vascular resistance ratio), were also determined. Pearson correlation coefficients were calculated between PVRi and Ea, Emax, and Ea/Emax. Receiving operating characteristic (ROC) curve analysis determined the diagnostic value of Ea/Emax in predicting vascular reactivity.

## Results

**Figure 1:**
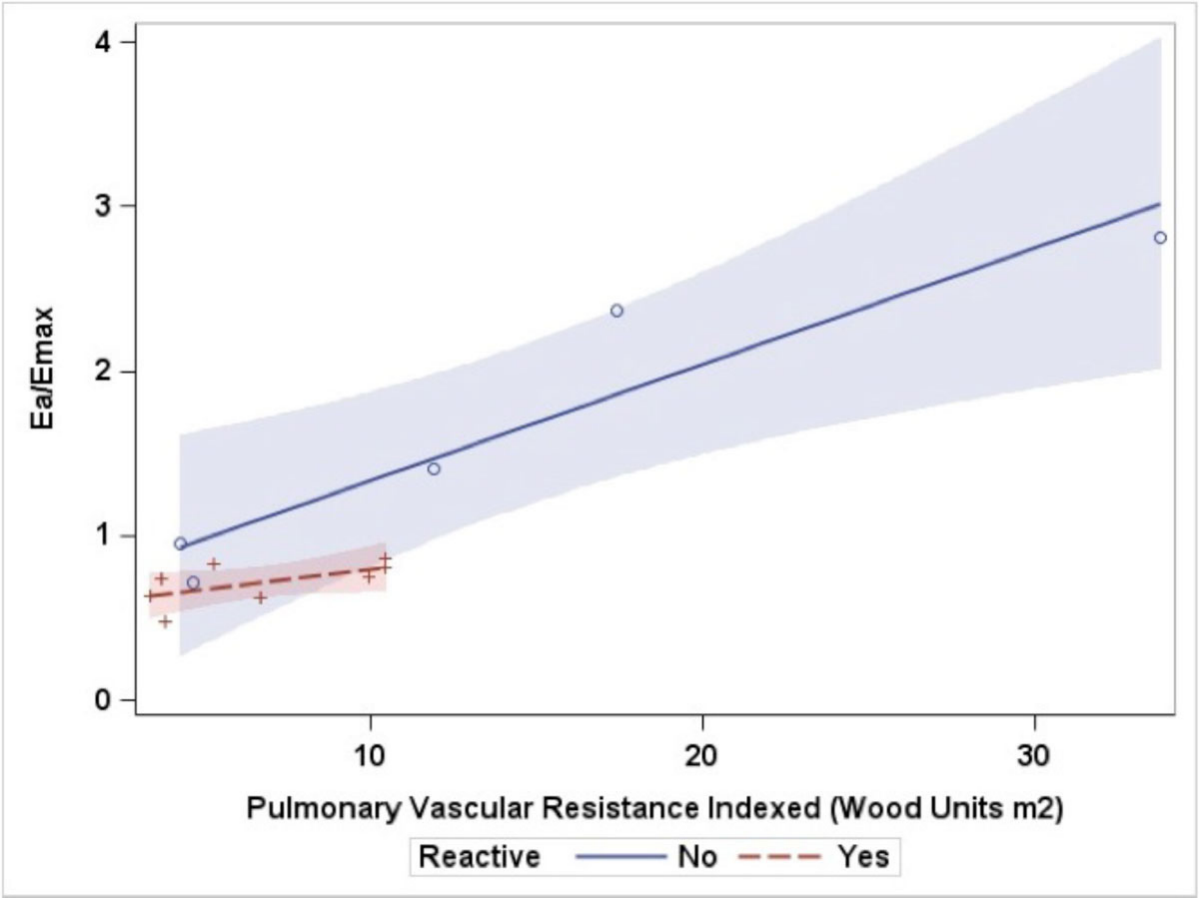
**Regression of ventricular arterial coupling ratio (Ea/Emax) and pulmonary vascular resistance indexed by reactivity**. The shaded areas represent the 95% confidence interval for each regression line. The lines depict different trajectories based on reactivity, which approached, but not reach statistical significance (p > 0.05).

**Figure 2:**
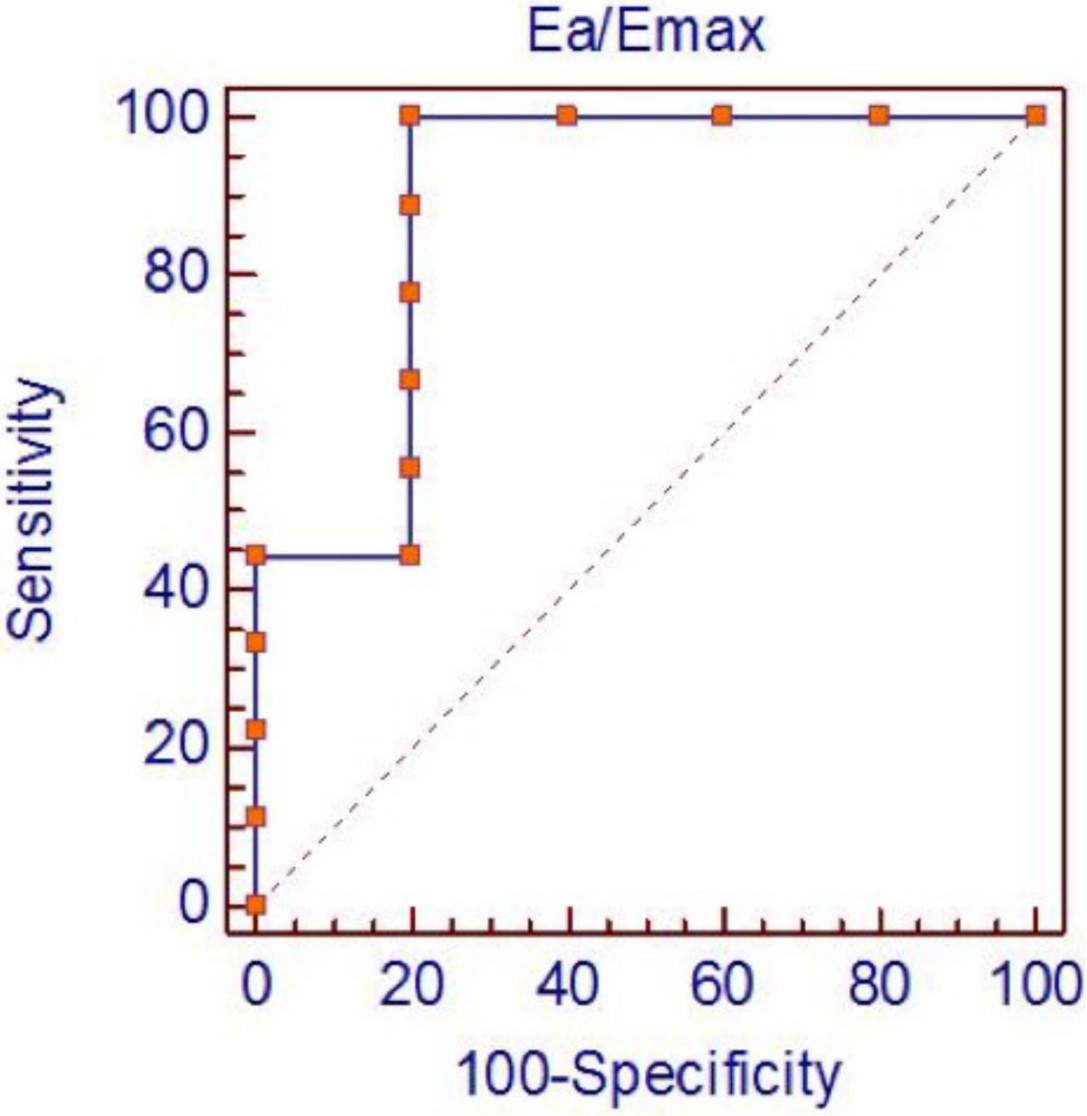
**Receiver operating characteristic curve demonstrating an optimal threshold Ea/Emax ratio of 0.85**. Using this criterion, is associated with a sensitivity of 100% and a specificity of 80%.

Sixteen subjects were identified for inclusion with equal gender distributions. Age ranged from 3 months to 23 years (mean 11.3+7.4 years). Ea and Ea/Emax increased with increasing severity defined by PVRi, with p < 0.001 for both. Ea/Emax (range 0.43-2.82) was highly correlated with PVRi (r = 0.92, 95% CI 0.79-0.97, p < 0.0001). Non-invasively derived ventricular arterial coupling was found to be significantly correlated with PVRi (r = 0.85, 95% CI 0.62-0.95, p < 0.0001), but with a lower correlation coefficient than with Ea/Emax derived from combined hemodynamic and CMR data. Regression of Ea/Emax and PVRi demonstrated differing lines when separated by reactivity, however, the lines were not significantly different (Figure 1). ROC curve analysis (Figure 2) revealed high accuracy of the Ea/Emax ratio in determining vascular reactivity. Ea/Emax of 0.85 had a sensitivity of 100% and a specificity of 80%. The area under the curve is 0.89 (p = 0.008), suggesting good discrimination between those who were and were not reactive.

## Conclusions

Measurement of ventricular arterial coupling, Ea/Emax, in pediatrics is feasible. Pulmonary vascular non-reactivity may be due to ventricular-arterial decoupling in which ventricular contractility fails to parallel increasing afterload in severe PAH. Use of Ea/Emax may have significant prognostic implication.

## Funding

This work was supported by UL1 TR000154 from NCATS/NIH, 5R01HL114753 from NHLBI/NIH, as well as K25-094749 and K24-081506.

